# Acute colonic flexures: the basis for developing an artificial intelligence-based tool for predicting the course of colonoscopy

**DOI:** 10.1007/s12565-022-00681-8

**Published:** 2022-09-02

**Authors:** Slawomir Wozniak, Aleksander Pawlus, Joanna Grzelak, Slawomir Chobotow, Friedrich Paulsen, Cyprian Olchowy, Urszula Zaleska-Dorobisz

**Affiliations:** 1 Department of Human Morphology and Embryology, Division of Anatomy, Wroclaw Medical University, Lower Silesia, Chalubinskiego 6a, Wroclaw, Poland; 2 Department of General Radiology, Provincial Specialist Hospital, Iwaszkiewicza 5, Legnica, Poland; 3 Friedrich Alexander University Erlangen-Nurnberg (FAU), Institute of Functional and Clinical Anatomy, Universtatsstr. 19, Erlangen, Germany; 4 Department of Oral Surgery, Wroclaw Medical University, Krakowska 26, Wroclaw, Poland; 5 Department of General and Paediatric Radiology, Wroclaw Medical University, M. Curie-Sklodowskiej 68, Wroclaw, Poland

**Keywords:** Colon, Colon flexures, CT scans, Artificial intelligence

## Abstract

Tortuosity of the colon is an important parameter for predicting the course of colonoscopy. Computed tomography scans of the abdominal cavity were performed in 224 (94 female, 130 male) adult subjects. The number of acute (angle not exceeding 90°) bends between adjacent colonic segments was noted and analyzed. Data were analyzed for correlation with gender, age, height and weight. An artificial intelligence algorithm was proposed to predict the course of colonoscopy. We determined the number of acute flexions in females to be 9.74 ± 2.5 (min–max: 4–15) and in males to be 8.7 ± 2.75 (min–max: 4–20). In addition, more acute flexions were found in women than in men and in older women (after 60 years) and men (after 80 years) than in younger ones. We found the greatest variability in the number of acute flexures in the sigmoid colon (0–9), but no correlation was found between the number of acute flexures and age, gender, height or BMI. In the transverse colon, older and female subjects had more flexures than younger and male subjects, respectively. Older subjects had more acute flexures in the descending colon than younger subjects. There are opportunities to use the number of acute flexures (4–7, 8–12, more than 12 flexures) to classify patients into appropriate risk categories for future incomplete colonoscopy. On this basis, we predicted troublesome colonoscopies in 14.9% female and in 6.1% male subjects.

## Introduction

The colon consists of intestinal segments attached to the abdominal wall or suspended from mesenteries in the abdominal cavity. The colonic flexures (other names: bendings, bends, flexions or angulations—defined as focal angle) are located between the segments and form its tortuosity. They are called acute (or sharp) when they bend at an angle of 90° or less. Other bends (more than 90°) are termed obtuse (Wozniak et al. 2019). The number of acute colonic flexures was determined to be 9–11 (range 5–19) (Eickhoff et al. 2010; Hanson et al. 2007; Khashab et al. 2009). The number of flexures correlated with difficulty in performing colonoscopy, but no further analyses were performed on correlations with other patient parameters. Based on the known anatomic description, we expect four or five flexures with acute angles. The colon can be examined by imaging methods, including computed tomography (CT).

CT is used for follow-up of various viscera, including the intestine (Cai et al. 2013; Carvalho et al. 2018). This investigation is a good platform for the development of other models that can be used for a new modern technology, for example based on artificial intelligence (AI) tools.

It has recently been shown that AI can be developed and applied for colonoscopy or wireless capsule endoscopy (Aoki et al. 2019). It has been used to detect and characterize polyps, mucosal healing, and inflammatory bowel dysplasia during colonoscopy. It can also be used to assess the quality of bowel preparation before the examination (Gubatan et al. 2021; Yang and Bang 2019; Yoo et al. 2021).

The aim of this study was to analyze the acute colon flexures and, on this basis, create a simple algorithm that can be used in the development of an AI system to classify patients according to predicted difficulty of colonoscopy. We hypothesized that there were a difference between patients’ anthropometric parameters and number of colon flexures which could interfere with the endoscopic examination course.

## Materials and methods

### Ethical issues

The provisions of the 1964 WMA Declaration of Helsinki (with further revisions) and Good Clinical Practice were the basis for conducting of our retrospective study (a post hoc design). No informed consent was obtained from patients to participate in this study. The project design was approved by the local ethics committee of the Medical University of Wroclaw (Decision No. 860/2019). The examinations were performed in 2018. The same group of patients was used for the analysis of the position of the descending-sigmoid flexure in the abdominal cavity (Wozniak et al. 2022).

### Patients, examiners, CT equipment and image analysis

CTs were performed in 224 patients: 94 female (F), 130 male (M) fasting (at least 4 h before examination) adult patients at the Provincial Specialist Hospital in Legnica, Poland. Patients were eligible for the study if they were aged at least 20. We established the border between younger and older patent as 60 years. Only patients without abdominal or pelvic surgery or colonic disease were included. Most patients (60%) were referred for diagnostics because of abdominal pain. Most subjects were 50–60 years old and had a body mass index (BMI) of 25–35 (significantly higher in M than in F). The detailed characteristics of the subjects are shown in Table 1.

**Table 1:** Subjects’ characteristics

Subjects’ characteristic	Values
Total number (F, M)	224 (F 94; M 130)
Age mean ± sd; years (range)	59.6 ± 13.4 (21–86)
BMI mean ± sd (range)	28.6 ± 5.7 (18.4–59.5)
Weight mean ± sd (range)	81.7 ± 17.8 kg (47–160)
Height mean ± sd (range)	168.8 ± 9.2 cm (148–198)

All the examinations were performed on Toshiba aquilion one 320-slice ct scanner (Toshiba Aquilion One, Toshiba Medical Systems, Tochigi Pref., Japan). Evaluations were obtained on non-contrast CT phase of abdomen and pelvis, with WW (window width): 400, WL (window level): 40 and 1 mm slice thickness. Parameters of CT: voltage = 120 kV, different current automatically adjusted by the scanner, depending on e.g. patients’ BMI. Two experienced radiologists (specialists with more than 10 years of experience in abdominal CT diagnosis) analyzed the data separately.

The number and localization of acute colonic flexures (angle not exceeding 90°) were examined. Subjects were examined in the supine position. All measurements were done by two independent radiologists on three-dimensional analysis of large intestine in separate calculations. Reconstructions were made with multiplanar reconstruction (MPR) to get simultaneous view of every dimension. The main statement to classify flexion as an acute was minimum 90 degrees in at least one dimension. Measurements tools (angles) of Vitrea (workstation VitreaExtend 6.7.4, TMS, Japan) were used in doubtful interpretation. All measurements were checked three times. The differences in final results were together discussed by radiologists to compromise the numbers.

### Statistical analysis

The data obtained were analyzed using the Statistica 13 software package (StatSoft Polska, Cracow, Poland). For categorical variables, results were summarized as *N* (%) and for continuous variables as mean ± standard deviation. Normality of variables was analyzed using the Kolmogorov–Smirnov test. For quantitative values, the *U* Mann–Whitney test and non-parametric ANOVA were applied, because normality was not found. For correlation between parameters, the Pearson coefficient, *r*-Spearmann test, multiple regression, and *χ*^2^ test were applied. All calculations were performed at a significance level of *p* = 0.05.

### Repeatability and reproducibility

In the preliminary study, we repeated the subjects’ assessments in 20 patients (analyzed by two experienced radiologists). The number of bendings was detected and analyzed in all trials, with no differences between investigators.

## Results

**Fig. 1:**
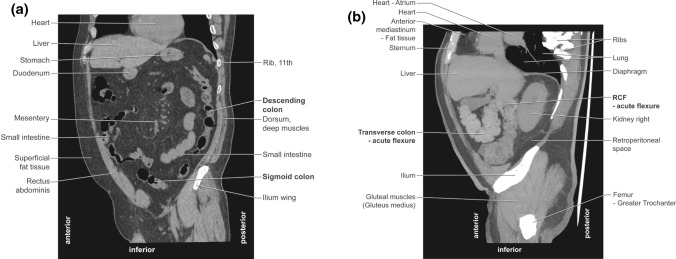
CT scans (tracing—main features). Subject with 4 acute flexures of the colon. Patient BW. F, age 69 years, weight 60 kg, height 164 cm, BMI 22.3. Date of examination: 18.08.2018. **A** Sagittal plane. Very straight course of the colon. No acute flexures on the scan. **B** Sagittal plane. Two acute colon flexures are shown on the scan (1 in the RCF, 1 in the transverse colon)

**Fig. 2:**
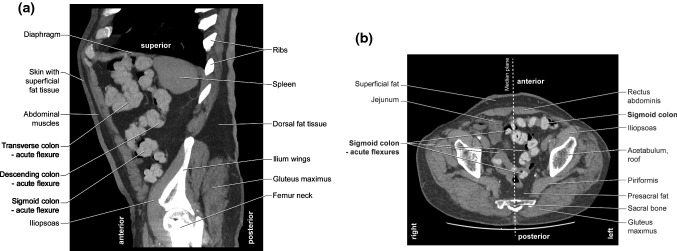
CT scans (tracing—main features). Number of acute colon flexions—20. Patient RW. M, age 83 years, weight 92 kg, height 178 cm, BMI 29. Date of examination: 30.11.2018. **A** Sagittal plane. There are 3 acute colonic flexures on the scan (1 in the sigmoid colon, 1 in the descending colon, and 1 in the transverse colon) **B** Horizontal plane—focused on the acute flexures in the sigmoid colon (4 flexures are seen on this scan)

We determined the number of acute flexions to be 9.74 ± 2.5 (min–max: 4–15) and 8.7 ± 2.75 (min–max: 4–20) in F and M, respectively. The detailed results are shown in Table 2. The influence of female gender and age. The differences between the number of acute bendings and gender were statistically significant (*U* Mann–Whitney test; *p* = 0.0007, *Z* = 3.36). In addition, more acute flexions were found in older women than in younger ones (as revealed by the multiple regression test; *p* < 0.05). Moreover, the females after the age of 60, and males after the age 80 had more acute flexions (Anova test F *p* = 0.009; M *p* = 0.008; post hoc test F *p* = 0.045; M *p* = 0.0). The detailed results are shown in Table 3. The influence of BMI. We found no statistically significant differences between the number of acute flexions and BMI in either sex (determined by Pearson’s correlation test, *r* = − 0.189 for F; *r* = − 0.01 for M; *p* ˃ 0.05 for both sexes). The CT images of patients with minimum (4) and maximum (20) acute colon flexions are shown in Fig. 1A, B and Fig. 2A, B, respectively. The influence of height. We found no statistically significant differences between the number of acute flexions and height in either sex (determined by Pearson’s correlation test, *r* = − 0.076 for F *r* = − 0.034 for M *p* ˃ 0.05 for both sexes.

**Table 2:** Acute bendings in the large intestine

Number of acute bendings (range)	Female (94) *N* (%)	Male (130) *N* (%)	Chi-square test *p* value (value)
In ascending colon (0–2)			
0	80 (85.1%)	116 (89.2%)	0.12 (4.3)
1–2	14 (14.9%)	14 (10.8%)	
In transverse colon (0–7)			
0	7 (7.5%)	29 (22.3%)	**0.001 (23.4)**
1–2	43 (45.7%)	68 (52.3%)	
3	25 (26.6%)	23 (17.7%)	
4 or more	19 (20.2%)	10 (7.7%)	
In descending colon (0–5)			
0	48 (51.1%)	68 (52.3%)	0.8 (1.7)
1–2	43 (45.7%)	58 (44.6%)	
3 or more	3 (3.2%)	4 (3.1%)	
In sigmoid colon (0–9)			
0	2 (2.1%)	3 (2.3%)	0.1 (14.1)
1–2	22(23.4%)	54 (41.6%)	
3–4	53 (56.4%)	51 (39.2%)	
5 or more	17 (18.1%)	22 (16.9%)	
In rectum (0)			
0	94 (100%)	130 (100%)	n/a

Statistically significant *p* value are shown in bold

*n/a* not applicable

**Table 3:** Number of acute colon flexures by gender and age (20 yrs range)

Age: years	Number of colon flexures: mean ± sd	
	94 Females (no) *p* = 0.009*	13 Males (no) *p* = 0.008*
20,00–39,99	7.8 ± 0.7 (12)	7.9 ± 0.7 (14)
40,00–59,99	9.9 ± 0.4 (32)	8.7 ± 0.5 (30)
60,00–79,99	9.9 ± 0.4 (46)**	8.6 ± 0.3 (81)
80,00–99,99	12.3 ± 1.2 (4)**	12.6 ± 1.2 (5)**

*One-way Anova; **post hoc test (F 60–79.99 yrs *p* = 0.045; 80–99.99 yrs *p* = 0.01. M 80–99.99 yrs, *p* = 0.0. Statistically significant *p* value are shown in bold

The analysis of consecutive colon parts: ascending colon. Most subjects (87.5%) had no acute flexures, 28 (12.5%) had 1–2 acute bendings of the ascending colon (1 flexure in 14 F and 11 M; 2 flexures in 0 F, 3 M). There were no correlations between the number of flexions and sex, age, or BMI. Transverse colon. No acute flexures were present in 36 subjects (7 F, 29 M). The largest subgroup (71%) had 1–3 acute flexions. In 1 male, we found 7 acute angulations. There were statistically significant correlations of the number of bends with female gender (*χ*^2^ test 23.4, *p* = 0.001) and the age of subjects (*r*-Spearmann test, *r* = 0.16; *p* < 0.05). No statistically significant correlation was found between the number of sharp transverse colon angulations and BMI. Descending colon. Flexures were not present in 116 (51.8%) subjects. We detected 1 flexure in 72 (32.1%) and 2 in 29 individuals (12.9%). There was a statistically significant correlation with age (*r*-Spearmann test, *r* = 0.16, *p* < 0.05). Sigmoid colon. The sigmoid was the part of the colon with the greatest variability—the number of acute flexures ranged from 0 to 9. Subjects with 2–4 acute bendings formed the largest subgroup numbering 156 (69.6%) individuals. No correlations were found between the number of acute bendings in the sigmoid colon and sex, age, or BMI. Rectum. No acute flexures were present in analyzed subjects.

The analysis of consecutive large intestine flexures: The right colic flexure was acute in 220 patients and obtuse in 4 M (3.1%). The left colic flexure was acute in all subjects. The descending-sigmoid flexure was acute in 69 subjects (26 F, 43 M). There were no correlations between acute descending-sigmoid flexure and age, sex, or BMI. Acute sigmoid-rectal flexures were found in 208 subjects (92.9%). This flexure was obtuse in 16 patients (5 F, 11 M). There were no correlations between acute sigmoid-rectal flexure and age, sex, or BMI. The detailed results are shown in Table 4.

**Table 4:** Acute large intestine flexures

The name of large intestine flexure	Female (94)	Male (130)
	Number of acute bendings (%)	
Hepatic flexure	94 (100%)	126 (96.9%)
Splenic flexure	94 (100%)	130 (100%)
Descending-sigmoid flexure	26 (27.7%)	43 (33.1%)
Sigmoid-rectal flexure	89 (94.7%)	119 (91.5%)

**Fig. 3:**
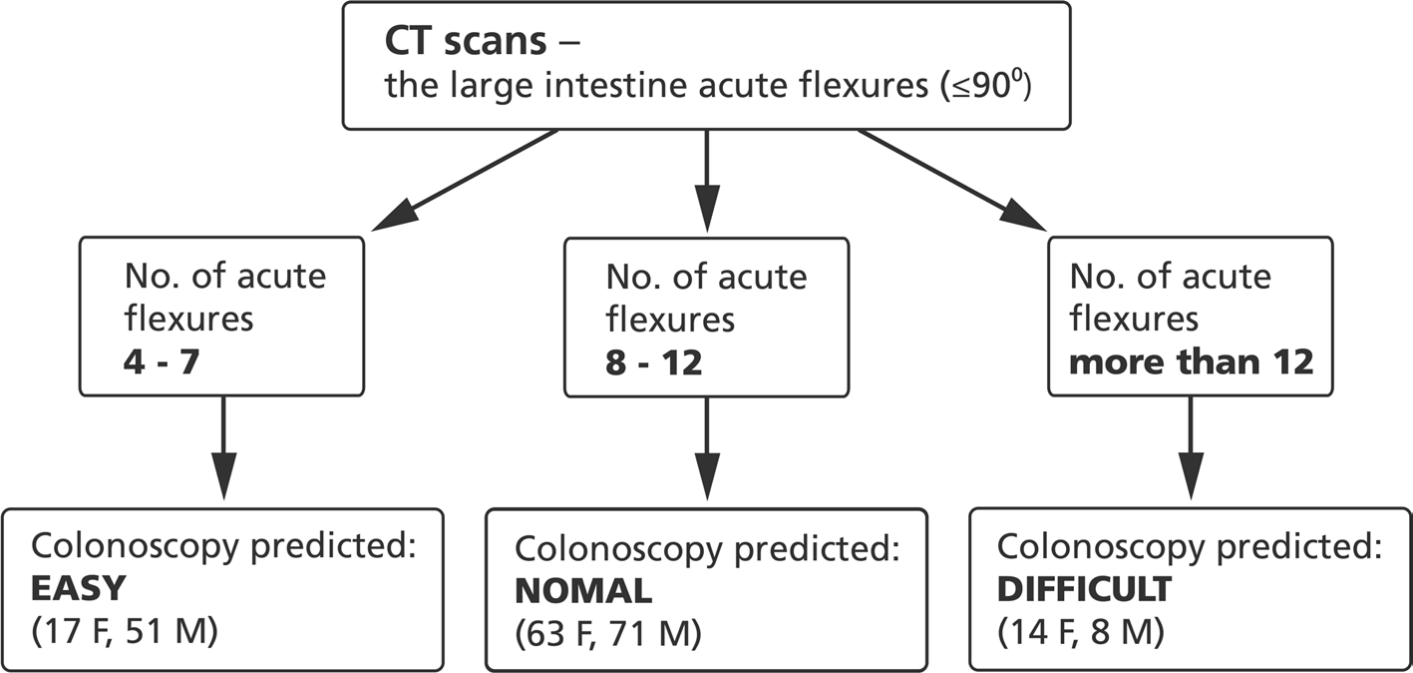
Overview of the natural language processing pipeline. Inputs are CT scans and the number of flexures. An output is a recommendation for a colonoscopy unit. “Easy” means that endoscopic examination can be performed by a specialist who is not experienced and works under supervision in a basic endoscopic unit. “Normal” means that colonoscopy can be performed in a basic or advanced endoscopic unit by a moderately experienced specialist. “Difficult” colonoscopy should be performed in the most advanced colonoscopic centers (by the most experienced specialist; possibly under general anesthesia)

The data obtained were used to divide the patient group into 3 subgroups with 4–7 (17 F, 51 M), 8–12 (63 F, 71 M), and more than 12 (14 F, 8 M) acute colonic flexions. The algorithm based on these results is shown in Fig. 3.

## Discussion

The morphology of the colon seems to be very well known (Abe et al. 2018). Currently, we can use modern methods, including CT colonography or magnetic resonance imaging, to analyze intestinal morphology (Cai et al. 2013; Carvalho et al. 2018). These studies allow a three-dimensional analysis of the viscera, which is a major advantage over other methods. In previous studies, authors focused on the length of the colon (Hounnou et al. 2002; Khashab et al. 2009) or the number of additional colon loops (Hanson et al. 2007; Eickhoff et al. 2010). Very little attention has been paid to the analysis of acute bowel bendings in individuals of different age, weight or age.

We found great variability in the morphology of the sigmoid and transverse colon. We supported the observations of other authors (Madiba et al. 2008; Raahave 2018; Bourgouin et al. 2012), which are important for different diseases or pathological bowel conditions. Utano et al. (321 patients studied with CT colonography) analyzed colon length in relation to gender and bowel habits (constipation) and found that total colon length was greater in women with constipation (infrequent bowel movements) (Utano et al. 2021). The same factors related to bowel anatomy are also important in diagnostic procedures, including colonoscopy or capsule endoscopy.

Bourgouin et al. collected anatomical descriptions of the colon useful for the reconstruction of different anatomical colon models (Bourgouin et al. 2012). We have summarized some additional data that can be used for this purpose. The bendings can be key factors for the introduction and the next step of the development of AI methods for the analysis of CT scans. We introduced the first, basic step of forming a computer-aided diagnosis system to separate patients with a potentially difficult and problematic colonoscopy examination. We set the number of acute bendings at 8–12, according to previous observations. Hanson et al. (study method: 200 patients; complete vs. incomplete colonoscopy; CT colonography) found 5–19 acute flexures per patient (Hanson et al. 2007). On average, more than two additional acute flexures were found in patients with incomplete colonoscopy. The increased number of sharp flexures correlated with the number of incomplete colonoscopies. Khashab et al. (study method: 505 patients; CT colonography) reported the mean number of acute bends to be 10.9 ± 2.4, with a maximum number of bends in the transverse colon of 4.7 ± 1.2 and in the sigmoid colon of 4.4 ± 1.2. No further analysis was performed (Khashab et al. 2009). Eickhoff et al. (study method: 200 patients; CT colonography and colonoscopies) determined a mean number of 9.6 ± 2.4 (Eickhoff et al. 2010). Alazmani et al. (study method: 24 patients; CT colonography) found that the transverse colon and sigmoid colon were the most tortuous sections of the colon; moreover, tortuosity was greater in the prone position than in the supine position (Alazmani et al. 2016). We found that women had more acute tortuosity than men; the colon is more tortuous in older women. Both older women and men had more acute bends in the transverse and descending colon. The sigmoid colon showed the greatest variability in the number of acute bends. We found 9.7 ± 2.5 (range 4–15) acute bends in our female subjects and 8.7 ± 2.75 (range 4–20) in our male subjects. Females have more acute flexions than males, but the cause for this is unknown. We speculate that maybe constipation is one of the reason for this phenomenon. In the same abdominal space longer intestine is lodged, so the colon is more tortuous. The more difficult colonoscopy course in female is multifactorial: the acute flexions can be only one of the factors. Saunders et al. (study method: 2194 colonoscopies) stated that reason for this may be in part to an inherently longer colon, especially transverse colon despite women’s smaller stature (Saunders et al. 1996).

No correlation was found between BMI and the number of acute flexions. Our patients were examined in the supine position.

AI is a method for processing data obtained during investigations. This tool is based on human intelligence capabilities derived from machine learning—creating mathematical algorithms from data and making decisions without human assistance—and deep learning (processing a large amount of information and creating algorithms to deal with key factors by linking them) (Deo 2015; Lee et al. 2017; Yang and Bang 2019). Its application in colonoscopy has great potential, and has already been applied to detect polyps and their analysis (Hoerter et al. 2020; Min et al. 2019). In our study, we introduced and described a tool that uses natural language processing techniques in an automated pipeline to synthesize information from CT scans and divide the studied patients into different clusters based on a set of acute colonic flexures. Our observations can be used as a reference range for classifying patients undergoing CT examination into different groups for predicting the course of colonoscopy—as shown in Fig. 3.

We hypothesize that this selection process will allow to separate patients with difficult colons (tortuosity) to perform colonoscopy in more experienced units with experienced endoscopists. The finding of a small number of sharp bendings can be useful to refer patients for colonoscopy in units that are not very experienced. The inclusion of this simple system can facilitate and enable a colonoscopic qualification system. If the number of acute colon flexions is detected during routine CT examination, specially designed computer systems could counsel patients about their risk of incomplete endoscopy and the recommended choice of a coloscopy lab for potential future colonoscopies. This would not require human monitoring. We have seen the usefulness of determining patients’ different bowel morphologies in separating those with predictable colonoscopy difficulties (more flexures) from those with likelihood of having an uncomplicated endoscopy. According to this rule, a subset of our subjects comprising 14 F (14.9%) and 8 M (6.1%) should be referred to have colonoscopy performed in an advanced endoscopic unit.

In further studies, researchers could focus on measuring correlations between the number of bowel flexures and segmental and total colon length. These automatically extracted results, describing colon morphology, would allow patients to be classified in appropriate risk categories of colon problems and be scheduled for appropriate monitoring intervals. We have noted the utility of this potential model in disease prevention health programs-particularly in patients with constipation or short bowel syndromes or as part of a colonoscopy qualification program. This initial classification of patients with a low or high risk of difficult colonoscopy could serve to reduce the number of incomplete colonoscopies performed in less experienced labs or by less experienced specialists. This would help reduce the number of repeat colonoscopies, saving money and increasing the number of successful endoscopies. Currently, the process for identifying high risk of incomplete colonoscopies is inefficient or does not exist at all.

We hope that this publication is a small step toward a recommendation algorithm that classifies patients as having a high or low risk for incomplete colonoscopy.

## Limitations

This study is not without limitations and has several acknowledged ones. First, the design of this study is post hoc—we used data obtained previously. Second, all patients were Caucasians, from one country region—this may introduce bias to the analysis. Third, colonoscopies were not performed on the control group to support our assumptions. Fourth, CT examinations were performed in the spine position, when colonoscopies in the right lateral decubitus position—small differences may be caused by it.

## Conclusions

1. Acute colonic flexions were more frequent in women than in men; moreover, older women (after 60 years) and men (after 80 years) had more acute flexions than younger ones.
2. Transverse colon: older and female patients had more flexures than younger and male patients, respectively.
3. Older patients had more acute flexures in the descending colon than younger patients.
4. We found the greatest variability in the number of acute flexures in the sigmoid colon, but no correlation was found between the number of acute flexures and age, sex, or BMI.
5. We found our algorithm, based on CT scans of colon flexures, to be useful in reckoning the risk for future incomplete colonoscopy. Based on it, we predicted troublesome colonoscopy in 14.9% Female and in 6.1% Male.
